# “Rescue” ablation of electrical storm in arrhythmogenic right ventricular cardiomyopathy in pregnancy

**DOI:** 10.1186/1471-2261-13-58

**Published:** 2013-08-13

**Authors:** Sebastian Stec, Tomasz Krynski, Jakub Baran, Piotr Kulakowski

**Affiliations:** 1 Department of Cardiology, Postgraduate Medical School, Grochowski Hospital, Warsaw, Poland ul. Grenadierów 51/59, 04-073, Warsaw, Poland

## Abstract

**Background:**

Radiofrequency ablation (RFCA) became a treatment of choice in patients with recurrent ventricular tachycardia, ventricular fibrillation, and appropriate interventions of implanted cardioverter-defibrillator (ICD), however, electrical storm (ES) ablation in a pregnant woman has not yet been reported.

**Case presentation:**

We describe a case of a successful rescue ablation of recurrent ES in a 26-year-old Caucasian woman during her first pregnancy (23rd week). The arrhythmogenic right ventricular dysplasia/cardiomyopathy (ARVD/C) was diagnosed 3 years earlier and several drugs as well as 2 ablations failed to control recurrences of ventricular tachycardia. RFCA was performed on the day of the third electric storm. The use of electroanatomic mapping allowed very low X-ray exposure, and after applications in the right ventricular outflow tract, arrhythmia disappeared. Three months after ablation, a healthy girl was delivered without any complications. During twelve-month follow-up there was no recurrence of ventricular tachycardia or ICD interventions.

**Conclusions:**

This case documents the first successful RFCA during ES due to recurrent unstable ventricular arrhythmias in a patient with ARVD/C in pregnancy. Current guidelines recommend metoprolol, sotalol and intravenous amiodarone for prevention of recurrent ventricular tachycardia in pregnancy, however, RFCA should be considered as a therapeutic option in selected cases. The use of 3D navigating system and near zero X-ray approach is associated with minimal radiation exposure for mother and fetus as well as low risk of procedural complication.

## Background

Radiofrequency ablation (RFCA) became a treatment of choice in patients with recurrent ventricular tachycardia (VT) and ventricular fibrillation (VF), and appropriate interventions of implanted cardioverter-defibrillator (ICD). Frequent recurrences of VT/VF triggering ICD discharges (electrical storm – ES) are a life threatening conditions, and particularly difficult to treat in a pregnant woman [1]. There are, however, no reports on rescue RFCA for electrical storm (ES) during pregnancy.

## Case presentation

In this paper, we describe a case of recurrent ES in a 26-year-old Caucasian woman during her first pregnancy (23rd week) with arrhythmogenic right ventricular dysplasia/cardiomyopathy (ARVD/C) diagnosed 3 years earlier. In 2008, the patient underwent ICD implantation and had 2 failed RFCA in the right ventricular outflow tract (RVOT) in another center for very frequent symptomatic premature ventricular complexes (PVCs) and VT.

During pregnancy, symptoms of PVCs and recurrent VT aggravated. She was unsuccessfully treated with a high dose of metoprolol followed by sotalol. The patient was diagnosed with posttraumatic stress disorder and treated with benzodiazepines. In July 2011, in the 23^rd^ week of pregnancy, 2 ES episodes with 10 appropriate shocks were documented and frequent recurrences of nonsustained VT were recorded despite conscious sedation (diazepam, 3 × 5 mg) and intravenous amiodarone. Therefore, the patient was transferred to our center by air ambulance for rescue RFCA.

**Figure 1:**
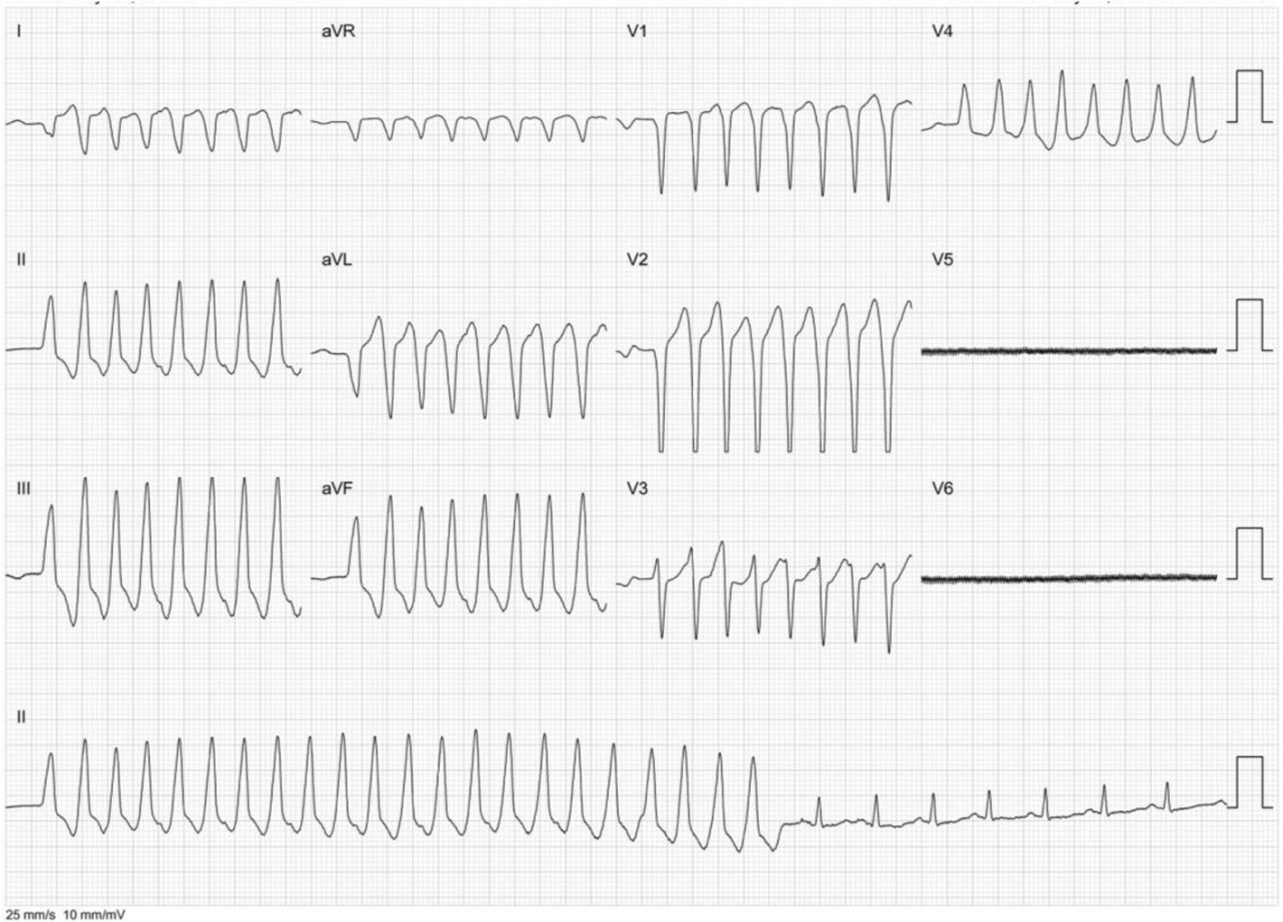
**Clinical PVC trigger of non-sustained VT in pregnant woman prior to intracardiac mapping (Lead V5 and V6 are disconnected).** Slight different morphology of PVC (first QRS) as compared to VT can be noticed.

**Figure 2:**
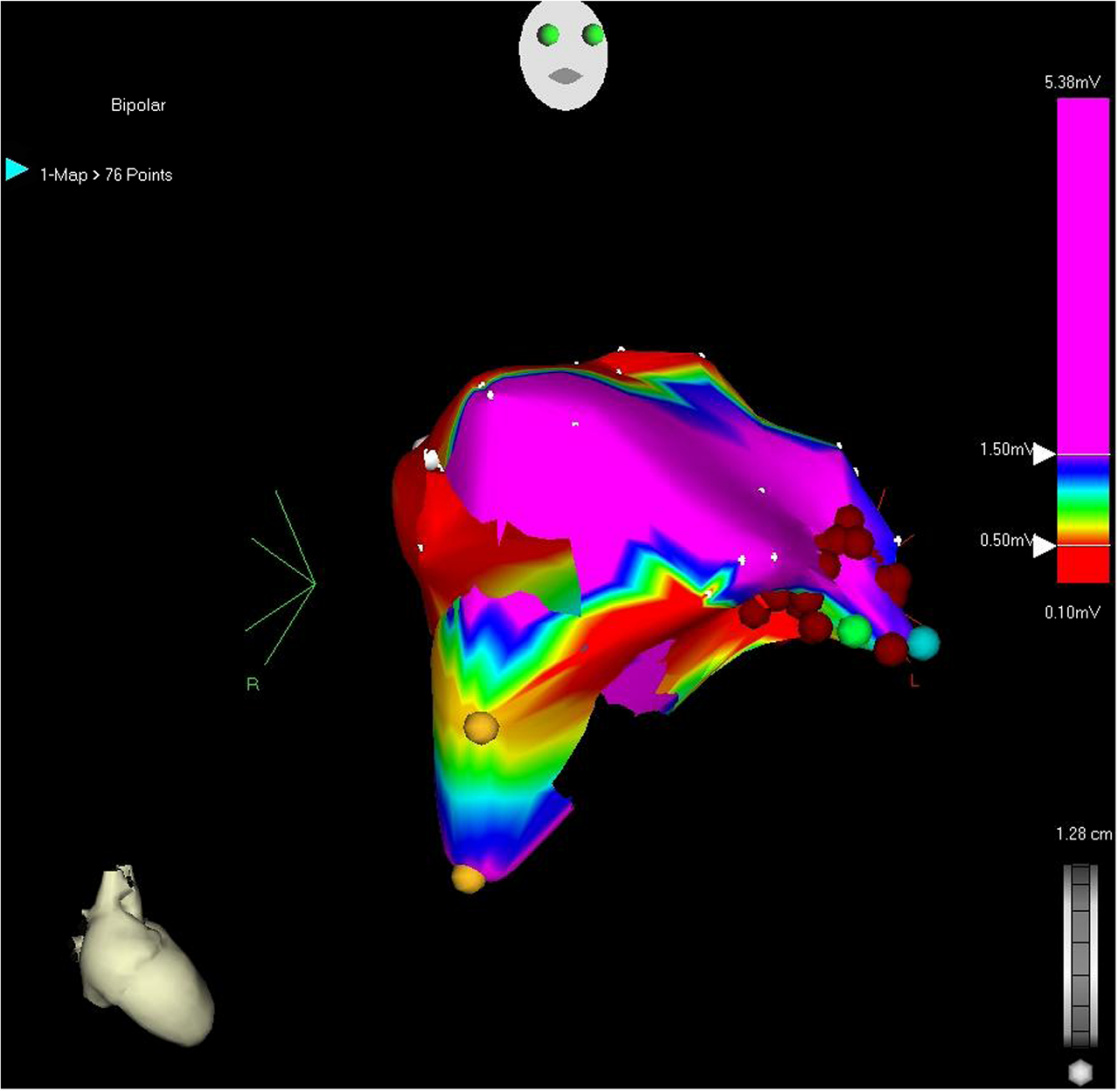
**Low density, simplified electroanatomical mapping of RVOT (52 points).** Yellow dot represent His position. Blue dot represent position of catheter where VT was located. Green dot represent point where PVC stopped and red dots represent additional bonus applications. Red area shows low voltage area (<1.5 mV).

**Figure 3:**
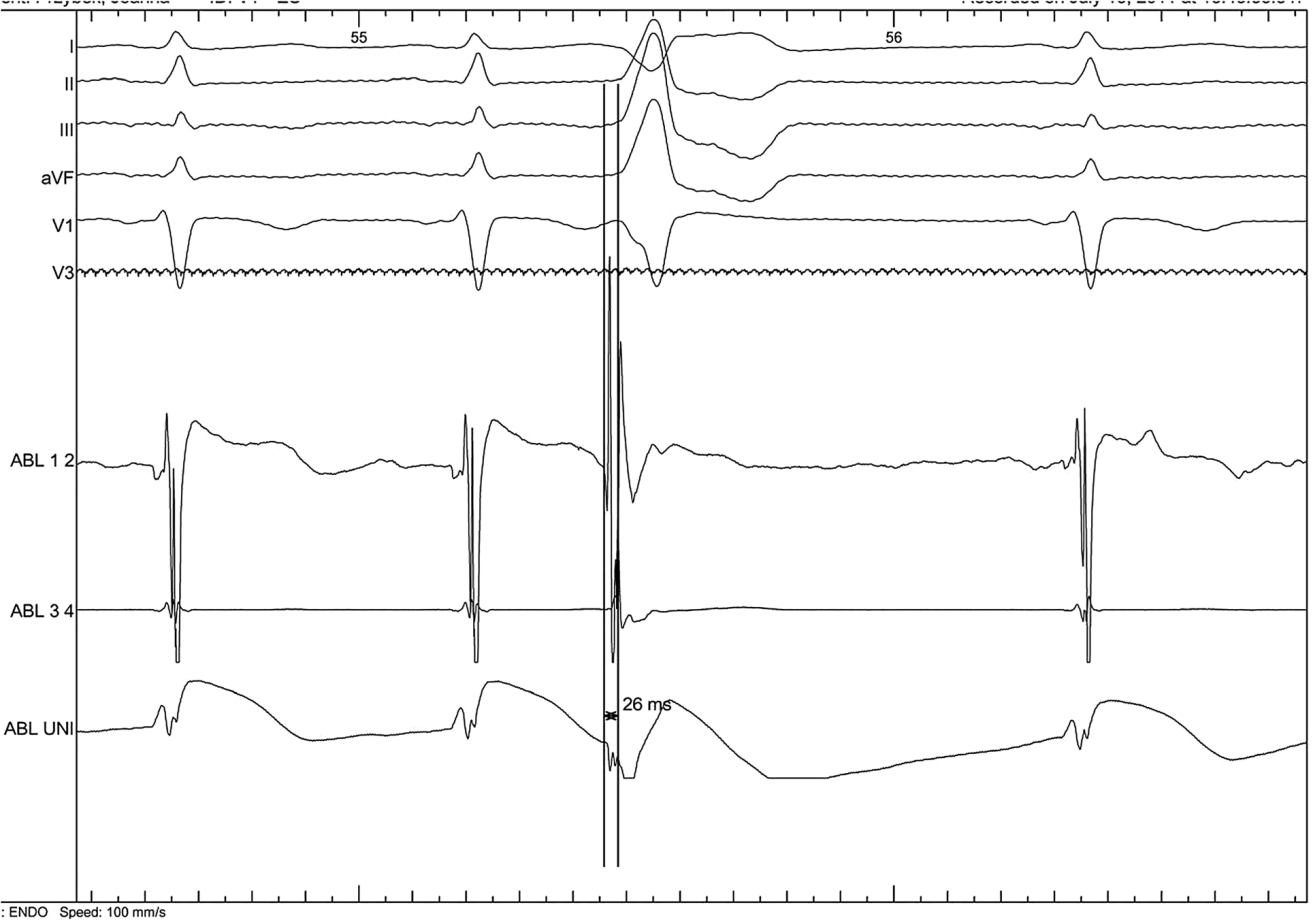
**Local activation mapping of PVC triggering fast VT from RVOT just before successful application.** Local signal from distal tip of ablation catheter proceeded QRS during PVC over 25 msek. ECG speed - 100 mm/s.

Just before the procedure, the patient had another ES with 3 adequate shocks due to fast VT up to 300 bpm triggered by focal PVCs with a slightly different morphology (Figure 1). Sedation was limited to 1 mg midanium intravenously. During mapping and ablation, frequent PVCs and PVCs-triggered nonsustained VT were recorded. A single 3.5-mm open-irrigation ablation catheter (Navistar) introduced from femoral approach and 3D mapping and navigation system (Biosense Webster, Bar Diamond, CA, USA) were used. After activation mapping (bipolar and unipolar) and pace mapping, PVC was located in the anterior part of RVOT with concordance with dipper location of VT focus (Figures 2 and 3).

Within 320 seconds and 10 applications, PVCs were no longer observed and VT were not recorded spontaneously, neither during programmed ventricular stimulation, including isoproterenol infusion. Total X-ray exposure time was 90 sec (dose: 10 Gycm2), mainly in order to confirm the position of ablation catheter and ICD lead.

The patient had no PVCs and recurrent VT within 48 hours of telemetry.Fetal examinations were normal prior and after RFCA. In the 36^th^ week of pregnancy, a healthy girl was delivered by caesarian section with 10 Apgar points. During 12 months of follow up, the patient had no ICD interventions and a Holter monitoring revealed no PVC. The patient was treated with a low dose of metoprolol for inadequate sinus tachycardia and antidepressant.

This case documents the first reported in literature, successful “rescue” RFCA during ES due to recurrent unstable ventricular arrhythmias in a patient with ARVD/C in pregnancy. RFCA procedures during pregnancy have been reported in single cases and very limited series of patients. They have been performed only in cases with unstable life-threatening supraventricular arrhythmias with excellent results and safety profile [2-6]. Current guidelines recommend metoprolol, sotalol and intravenous amiodarone for prevention of recurrent VT in pregnancy, however, RFCA should be considered as a therapeutic option in selected cases [1].

Hormonal or autonomic imbalance may be associated with the risk of ES in pregnancy [1,5,7]. The incidence of ES in patients with ARVD/C is reported to be 20%, that should require effective RFCA or adequate consulting before pregnancy [8,9] ARVD/C consisted of 14% of cases in a large single center registry of ES ablation in patients with organic heart disease and ICD. Successful RFCA with non-inducibility of any VT has been proven to be associated with very good outcome [10].

In conclusion, using 3D navigation system, near zero- X- ray approach and in the nearest future contact force measurements, even complex ventricular maternal arrhythmia may be treated during pregnancy. Moreover current progress in invasive electrophysiology may grant mother and fetus a minimal or complete elimination of radiation exposure during RFCA, efficient substrate mapping and a low risk of procedural complications.

## Conclusions

Improvement of RFCA with advanced modern technology should encourage the use of this method for treatment for recurrent life-threatening maternal ventricular arrhythmias or ES in pregnancy.

## Consent

Written informed consent was obtained from the patient for publication of this case report and any accompanying images. A copy of the written consent is available for review by the Editor-in-Chief of this journal.

## Competing interests

The authors declare that they have no competing interests.

## Authors' contributions

SS carried out ablation procedure and drafted the manuscript. TK was involved in ablation procedure and electroanatomical mapping with 3D mapping system. JB participated in ablation, worked with eloctrophysiological system and drafted the manuscript. PK participated in ablation also was involved in drafting the manuscript and revised it critically. All authors read and approved the final manuscript.

## Pre-publication history

The pre-publication history for this paper can be accessed here:

http://www.biomedcentral.com/1471-2261/13/58/prepub
